# The effects of cognitive reappraisal following retrieval-procedures designed to destabilize alcohol memories in high-risk drinkers

**DOI:** 10.1007/s00213-015-4164-y

**Published:** 2015-12-14

**Authors:** Tiffany Hon, Ravi K. Das, Sunjeev K. Kamboj

**Affiliations:** Clinical Psychopharmacology Unit, Research Department of Clinical, Educational and Health Psychology, University College London, London, WC1E 6BT UK

**Keywords:** Addiction, Alcohol, Reconsolidation, Memory, Reappraisal, Cognitive therapy, Behavioural therapy, Craving, Alcohol fluency

## Abstract

**Rationale:**

Addiction is a disorder of motivational learning and memory. Maladaptive motivational memories linking drug-associated stimuli to drug seeking are formed over hundreds of reinforcement trials and accompanied by aberrant neuroadaptation in the mesocorticolimbic reward system. Such memories are resistant to extinction. However, the discovery of retrieval-dependent memory plasticity has opened up the possibility of permanent modification of established (long-term) memories during ‘reconsolidation’.

**Objectives:**

Here, we investigate whether reappraisal of maladaptive alcohol cognitions performed after procedures designed to destabilize alcohol memory networks affected subsequent alcohol memory, craving, drinking and attentional bias.

**Methods:**

Forty-seven at-risk drinkers attended two sessions. On the first lab session, participants underwent one of two prediction error-generating procedures in which outcome expectancies were violated while retrieving alcohol memories (omission and value prediction error groups). Participants in a control group retrieved non-alcohol memories. Participants then reappraised personally relevant maladaptive alcohol memories and completed measures of reappraisal recall, alcohol verbal fluency and craving. Seven days later, they repeated these measures along with attentional bias assessment.

**Results:**

Omission prediction error (being unexpectedly prevented from drinking beer), but not a value prediction error (drinking unexpectedly bitter-tasting beer) or control procedure (drinking unexpectedly bitter orange juice), was associated with significant reductions in verbal fluency for positive alcohol-related words. No other statistically robust outcomes were detected.

**Conclusions:**

This study provides partial preliminary support for the idea that a common psychotherapeutic strategy used in the context of putative memory retrieval-destabilization can alter accessibility of alcohol semantic networks. Further research delineating the necessary and sufficient requirements for producing alterations in alcohol memory performance based on memory destabilization is still required.

## Introduction

Maladaptive memories and cognitions are at the heart of many psychiatric disorders. A central aim of existing psychological therapies is to reduce the propensity of these memories to result in harmful behaviours. Yet, long-established maladaptive memories are difficult to permanently modify and often re-emerge as a result of a variety of well-characterized relapse processes (e.g. reinstatement, renewal, spontaneous recovery) (Bouton 2002). Addiction and anxiety disorders are prototypical in this regard: they both centrally involve very strongly encoded associative memories that promote relapse when the relevant conditioned stimuli are encountered. In the case of psychological treatment, re-emergence of symptoms after treatment reflects the fact that current therapies do not permanently modify existing maladaptive memories. Instead, by introducing alternative representations (e.g. through extinction-based treatments), the influence of maladaptive memories is temporarily lessened because they are outcompeted by new associative representations. However, the survival of original maladaptive memories after treatment represents a continuing latent risk for relapse.

Under certain circumstances, however, retrieval of well-learned, cortically consolidated memories become ‘destabilized’ at the level of their putative synaptic representations (Nader et al. 2000; Przybyslawski et al. 1999), allowing direct experience-dependent updating of encoded associations (Monfils et al. 2009; Schiller et al. 2009). The updated memory is then restabilized, completing a cycle of memory ‘reconsolidation’. This is critical, as direct and permanent modification of maladaptive memories, rather than competitive inhibition, should greatly increase the durability of therapeutic effects (Soeter and Kindt 2010; Soeter and Kindt 2011). Indeed, it has recently been argued that the circumstances under which cognitive behavioural therapy (CBT) and emotion-focused therapies show long-lasting benefit arise from successful engagement of reconsolidation-updating mechanisms and direct ‘treatment’ of maladaptive memory (Lane et al. 2014).

In support of this hypothesis are two recent studies demonstrating impressive, long-lasting effects of single-session reconsolidation-based interventions. Xue and colleagues (2012) demonstrated reduced craving in heroin addicts, lasting for up to 6 months following an intervention involving cue exposure conducted within the ‘reconsolidation window’ following retrieval of maladaptive heroin-related memories. Soeter and Kindt showed complete transformation of fear in people with spider phobia following blockade of reconsolidation of spider-evoked fear memories with the β-blocker propranolol, with effects lasting for at least 1 year (Soeter and Kindt in press). These findings suggest that the modification of destabilized maladaptive memories may lend durability to subsequent treatment effects. If this hypothesis is correct, incorporating procedures involving the retrieval of maladaptive memory prior to intervention could be transformative for the efficacy of psychological and pharmacological therapies, requiring minimal additional cost or modification of existing therapies.

However, consolidated memories do not destabilize whenever they are retrieved. If they did, this could lead to ‘catastrophic interference’ effects (Eichenbaum 2006). Observed null findings in reconsolidation research may therefore reflect an ‘ordinary’ failure to destabilize memories upon retrieval (Piñeyro et al. 2014; Chan et al. 2010). Most animal behavioural studies on reconsolidation have focused on disrupting the simple associative learning that underlies fear or excessive appetitive drive states (addictive behaviour) by administering drugs during retrieval that have protein synthesis-disrupting effects. While these studies suggest that memory destabilization at retrieval is readily achieved in rodents by simply presenting an unreinforced conditioned stimulus, studies in humans have tended to highlight an additional necessary role for surprise or ‘prediction error’ (PE) in memory destabilization during retrieval (Sevenster et al. 2013a). This phenomenon is aligned with neurocomputational models of reinforcement learning (Sutton and Barto 1998; Sutton and Barto 1981; Schultz et al. 1997; Waelti et al. 2001), in that the resulting plasticity of long-term memory enables adaptive behavioural flexibility in the face of changing environmental circumstances.

A recent pharmacological study with dependent, quitting tobacco smokers which did not include an explicit PE during retrieval failed to show the expected pharmacologically induced disruption (via NMDA receptor inhibition with memantine) of reward memory during reconsolidation (Das et al. 2015b). However, another study in people with cocaine dependence showed evidence of reduced craving and cue reactivity following post-retrieval propranolol, despite the absence of explicit PE (Saladin et al. 2013). Procedural differences between these two studies may have determined whether simple non-reinforced presentation of conditioned stimuli during memory retrieval was sufficient to engender PE. In addition, it may be that only intermediate levels of destabilization are achieved using unreinforced conditioned stimuli in humans. In our recent study, the effectiveness with which appetitive memory value was overwritten following a counter-conditioning procedure seemed to vary with the intensity of PE (Das et al. 2015a). The clearest counter-conditioning response 1 week later was observed following an explicitly surprising retrieval procedure and a smaller response observed in the unreinforced conditioned stimulus condition.

If PE is indeed central to memory destabilization, it is important to identify conditions that can engender sufficient mismatch between expectation and outcome to render long-term memories labile and susceptible to modification. This is challenging because the nature and extent of expectancy violations required to destabilize memory when learning history is extensive and unknown (as is the case for the learning that underlies addictive disorders) is unclear. A different challenge is that the degree of mismatch between original learning and the retrieval experience must not be too large, as this will initiate new learning rather than destabilize existing memories (Osan et al. 2011).

In addition, the efficacy of behavioural and cognitive memory-modifying procedures applied within the reconsolidation window remains largely unknown. Although extinction through repeated unreinforced cue exposure and counter-conditioning following drug memory destabilization may be effective in experimental settings (Xue et al. 2012; Das et al. 2015a), they are not standard psychotherapeutic techniques and their efficacy and acceptability remain unclear. Moreover, the conditions under which beneficial effects are observed following ‘retrieval-extinction’ are unclear, and there may be circumstances when this approach can lead to enhanced reinstatement of drug seeking in animal models (Hutton-Bedbrook and McNally, 2013). Rather than these non-standard techniques, psychological treatment of substance use disorders often relies on motivational and emotion regulation procedures (Beck et al. 2011). In theory, once destabilized, memory networks should become ‘permeable’ to a variety of novel representations, including those produced by more conventional psychotherapeutic techniques.

In this study, we investigate the effects of such a technique—reappraisal of maladaptive alcohol-related cognitions—in high-risk drinkers during a period of putative memory destabilization. To achieve the latter, we omitted an expected outcome (beer drinking) during retrieval of alcohol memories (i.e. an omission-PE), a procedure we used in our previous study of counter-conditioning in at-risk drinkers (Das et al. 2015a). In addition, however, we also examined the effect of unexpectedly altering the value of the outcome (value-PE). This was achieved by making the beer taste extremely bitter and unpleasant. We tested these two different PE procedures because, as noted above, the nature and optimal level of novelty required for destabilization of appetitive memories remains unclear.

Since modulation of reconsolidation is expected to alter memory networks, our primary hypotheses and outcomes were memory-related. In particular, we examined the effects of the two PE-retrieval procedures (followed by reappraisal) on memory for the reappraisals themselves (i.e. participant-generated responses to ‘maladaptive’ alcohol-related appraisals) and on alcohol-related semantic memory. A recent model of reconsolidation-modulation-based psychological treatment suggests that maladaptive behaviour is supported by a tripartite cognitive-affective system comprising autobiographical memory, semantic structures and emotional responses (Lane et al. 2014). Modulation of maladaptive memory structures subserving excessive drinking behaviour may therefore be expected to be reflected in semantic memory performance (e.g. retrieval of alcohol-related words in a verbal fluency test). In line with the tripartite cognitive-affective system proposed by Lane and colleagues (Lane et al. 2014), we focused on fluency for valenced alcohol-related words in the current study. These associations are particularly relevant given that positive-negative is one of two dominant dimensions of alcohol semantic space (Kramer and Goldman 2003).

A primary goal of reappraisal as applied in CBT is to modify representations of rules and schemas encoded in semantic memory, such that the influence of unrealistic and maladaptively positive appraisals (e.g. ‘drinking gives me energy’) are downregulated, while negative expectancies (e.g. ‘alcohol makes me argumentative’) become more salient (Beck et al. 2011; Goldman 1994). As such, we hypothesized that one or both of the PE-retrieval groups would show a pattern of reduced accessibility of positive, and increased accessibility of negative, alcohol-related words. A previous study with abstinent opioid-addicted individuals showed memory impairment for valenced (positive and negative, but not neutral) heroin-related words following post-retrieval propranolol (Zhao et al. 2011). Generalized memory impairment for both positive and negative words may reflect relatively indiscriminate effects of a systemically administered pharmacological agent in contrast to what might be achieved using a more targeted emotion regulation technique.

It is reasonable to assume that positive effects of reappraisal on decision-making and behaviour following CBT depend on memory for these new competing representations (Harvey et al. 2014). As such, we sought to determine if reconsolidation-modulating procedures also improve memory for reappraisals generated to counteract maladaptive appraisals.

In addition to these memory-related outcomes, we assessed the effects of retrieval-PE-reappraisal on a number of secondary variables. For example, it is possible that changes in accessibility of alcohol memory networks following their modification during reconsolidation would be reflected in endorsement of declarative statements affirming a motivation to drink less and/or a recognition of harms. We tested this possibility using a measure of ‘readiness-to-change’ as a secondary measure. Other secondary measures included drinking behaviour, alcohol craving and attentional bias. The latter two constructs are weakly coupled metrics of latent maladaptive memory strength, and their reduction has potentially important clinical implications (Field and Cox 2008; Field et al. 2009). In addition, their inclusion allows a direct comparison with findings from our recent study (Das et al. 2015a), in which we found evidence for updating of alcohol memory across a variety of outcomes, including reductions in alcohol attentional bias. Those effects are best understood as resulting from associative-affective learning processes in a network of coupled affective and salience-related attributes (Stacy and Wiers 2010; Field et al. 2009). However, it is also possible that any procedure which effectively modifies alcohol memory networks during reconsolidation (including non-associative procedures, such as reappraisal) may produce these effects. Given the relatively early stage of research on modulation of appetitive memories during reconsolidation in humans, it seems legitimate to probe its effects using a variety of additional (non-memory) outcomes, including attentional bias, craving and drinking behaviour.

## Methods

### Participants

Participants were hazardous drinkers, defined as individuals who consumed alcohol in excess of UK Department of Health guidelines at least 4 days a week (>3 or 4 units of alcohol/day for men and women, respectively) and scored ≥8 on the Alcohol Use Disorders Identification Test (AUDIT). Inclusion criteria were (i) regular beer drinking and (ii) an interest in moderating drinking. Given that the aims of the study were to examine basic assumptions about reconsolidation and its treatment implications, it was important to test these in a clinically relevant sample in whom the potential risks for harmful effects were minimal. As such, exclusion criteria included a diagnosis of alcohol dependence, based on the Structured Clinical Interview of DSM-IV (SCID), and the presence of any self-declared medical or psychiatric conditions requiring treatment. Participants were randomly allocated to (i) omission-PE, (ii) value-PE or (iii) control condition. Initially, 48 participants were recruited (*n* = 16 per group). One participant (in the value-PE group) did not comply with experimental instructions and was excluded, leaving a total sample of 47 participants, each tested on two occasions. Participants received £20 for completing the study. The study received ethical approval from the University College London (UCL) Research Ethics Committee. Participants provided written informed consent in line with a procedure approved by the UCL ethics committee.

### Measures

#### Questionnaires

**Fig. 1:**
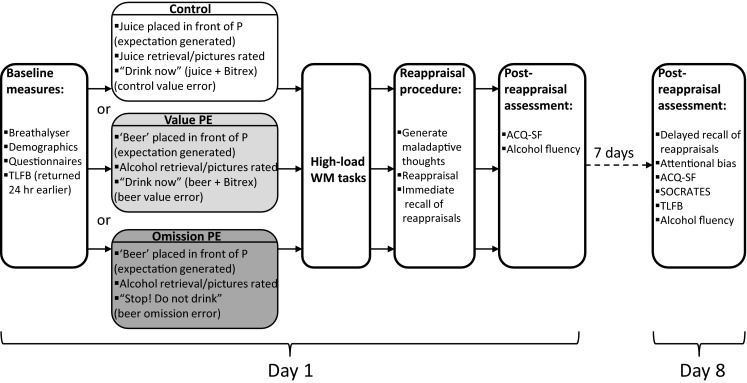
Study procedure over the course of two testing sessions

Assessments of baseline depressive symptoms, psychological mindedness and regulation of appetitive and aversive motives were measured using the Beck Depression Inventory (BDI; Beck et al. 1996), the Psychological Mindedness Scale (PM Scale; Conte et al. 1996) and Behavioural Inhibition and Activation Scales (BIS/BAS; Carver and White 1994), respectively, on the first testing session (day 1) prior to the reactivation procedure (Fig. 1). The Timeline Follow Back for Alcohol (TLFB; Sobell and Sobell 1992) assessed participants’ alcohol consumption in the week prior to the first testing session and 7 days later (day 8; see procedure below). Current craving for alcohol was assessed with the 12-item Alcohol Craving Questionnaire (ACQ-SF; Singleton et al. 1994), which is comprised of four subscales: expectancy, purposefulness, emotionality and compulsiveness. Readiness-to-change drinking habits were assessed using the Stages of Change Readiness and Treatment Eagerness Scale (SOCRATES; Miller and Tonigan 1996).

#### Alcohol verbal fluency

This task (Goldstein et al. 2007) assesses specific semantic fluency for alcohol-related words. Participants are instructed to name as many alcohol-related words as possible in 1 min. Responses were audio recorded and independently coded into three categories: neutral, positive and negative valence by two researchers. Repetitions and errors were excluded. Inter-rater reliability was assessed using two-way mixed, absolute agreement, average measures inter-class correlation coefficients (McGraw and Wong 1996): ICC = .986 for positive, .985 for negative and .998 for neutral words.

#### Attentional bias

Ten picture pairs were used that contained an alcohol-related (target) picture matched with a composition- and complexity-matched (non-target) picture that did not depict alcohol. The target pictures consisted of the four beer pictures used during memory reactivation, three novel pictures of beer and three novel pictures of wine, each always presented alongside its matched non-target. Wine and novel beer pictures were used to determine whether any effects on oculomotor attentional bias generalized to novel and less-preferred alcohol cues. In addition, there were eight pairs of neutral filler pictures from the IAPS database, selected on the basis of low arousal and neutral affect, in order to assess non-specific response biases. Each picture pair appeared for 2000 ms, after which the pictures disappeared and a triangular probe appeared in the location where one of the pictures had been. The triangle either pointed upwards or downwards, and participants had to respond to the orientation of the probe as quickly and accurately as possible on a keyboard. This behavioural response was purely to ensure maintained task engagement. Attentional bias scores were calculated as target picture dwell time minus matched control image dwell time. These were calculated after removing extreme responses (<100 ms after stimulus onset or >3 SDs from an individual’s mean).

All picture pairs were presented in a random order. Picture pairs were each presented eight times, counterbalanced for laterality of target picture (left or right), laterality of probe location (ipsilateral or contralateral to target picture) and probe orientation (pointing up or down). All pictures were 300 × 300 pixels.

Eye movements during the task were tracked with an Eyelink 1000 desktop-mounted eye tracker (SR Research, Ontario, Canada) with a sampling rate of 1 kHz. Participants’ heads were stabilized in a head mount set 60 cm away from the computer screen throughout.

#### Retrieval cues

Four prototypical beer pictures were selected to act as beer memory reactivation cues, as used in our previous study of retrieval-induced memory reactivation (Das et al. 2015a). These depicted beer taps on a bar, a poured pint of beer, an ice bucket filled with beer bottles and a can of beer being poured into a pint glass, representing the major modes of beer consumption. In the control group, orange juice pictures depicted an orange, a glass of orange juice being poured, a glass of poured orange juice and a woman consuming orange juice. The orange juice pictures were selected to be as visually and compositionally similar to the beer pictures as possible to minimize effects that were not specific to the reactivation manipulation. All stimuli were presented on a 1024 × 768 pixel 21-in. flat-screen monitor. In addition, in vivo retrieval cues were used: a chilled glass of beer or orange juice.

#### Memory reactivation

In both alcohol retrieval PE conditions, a 150-ml glass of chilled non-alcoholic beer was placed in front of participants during reactivation. Non-alcoholic beer was used to prevent alcohol-induced effects on performance, although to maintain appropriate alcohol expectancies, participants were not made aware that the drink did not contain alcohol. Bitrex (denatonium benzoate; 12 ml 0.067 %) was added to the beer in the value-PE group. This harmless and odourless additive is extremely bitter and made the beer highly aversive in this group. Participants were informed that after rating a set of beer pictures, they would be asked to consume the drink in front of them according to on-screen prompts. In the control condition, a 150-ml glass of chilled orange juice was used as an in vivo cue; participants rated the four orange juice pictures that served as control retrieval cues. Participants were asked to recall memories of typical (orange juice or alcohol) drinking episodes evoked by the pictures to guide their ratings on urge to drink and pleasantness using a 0–10 (not at all–extremely) scale.

Participants also rated (0–10) the expected pleasantness of the drink in front of them, after which on-screen prompts instruct participants to pick up the drink (prompt 1) and prepare to drink it (prompt 2). Participants in the control and value-PE groups also followed instructions to consume the drink (prompt 3) as expected. Participants in the omission-PE group followed the same first two prompts, but prompt 3 was ‘STOP! DO NOT DRINK’. These participants were required to put the drink down and alert the experimenter. Each prompt lasted 3000 ms. Participants in the control (juice) and value-PE groups then also rated the actual pleasantness of the drink they consumed.

#### Working memory (distractor) tasks

Distractor tasks began immediately after the retrieval procedure and included letter and category fluency tests, digit span forward and backward, a numeric and alphanumeric trail making and digit cancellation. These distractor tasks took approximately 10 min and were used to reduce the likelihood of maintenance of reactivated stimuli in working memory during cognitive restructuring, as this could bias learning towards new learning, rather than memory updating. Performance in the distractor tasks was not of primary interest to the current study and was therefore not reported.

#### Reappraisal

CBT involves identifying and challenging (reappraising) rules and beliefs that reside in semantic memory. As such, participants first identified personally relevant ‘maladaptive’ alcohol-related appraisals that they might experience before or during a drinking episode. To enable relatively rapid identification of such thoughts, participants were provided with a prompt sheet containing 22 example statements and asked to identify the ones that appeared most relevant to them. They were also encouraged to use wording that made the maladaptive appraisal personally relevant. They then generated ‘arguments’ (reappraisals) that challenged these maladaptive thoughts, as in standard CBT (Beck 2011). Again, a prompt sheet was provided to help participants identify relevant alternative thoughts. Both maladaptive appraisals and reappraisals (original and recalled) were recorded. Immediately after the reappraisal procedure, participants rated the ‘helpfulness’ and ‘ease’ of the reappraisal procedure on a 0–10 scale to determine engagement with the procedure.

Memory for these reappraisals was tested with cued recall on the first testing session (immediate recall, after ratings of helpfulness/ease) and 7 days later (delayed recall; see Fig. 1). Participants’ original maladaptive appraisals were used as cues for recall of their reappraisals. Each recalled reappraisal that was semantically and thematically consistent with the originally generated reappraisal scored 1, and 0 if not. Consistency between each participant’s original and recalled reappraisals was independently rated by two researchers. Inter-rater reliability was assessed using two-way mixed, absolute agreement, average measures ICC (McGraw and Wong 1996), which was in the excellent range ICC = 0.908. Discrepancies in the scoring of recalled reappraisals between raters were resolved through discussion. Percentage correct recall on the first session and 7 days later was calculated for each participant.

### Procedure

Participants were informed that the experiment involved examining attitudes towards drinking and would require them to consume a drink, which might be bitter-tasting. All participants completed two sessions, separated by 1 week (from here on, the first testing session is designated ‘day 1’ and the second, 7 days later, ‘day 8’). The day before the day 1 session, participants completed the TLFB and returned it via email. This was to minimize alcohol memory retrieval immediately prior to the retrieval manipulation on day 1. Both sessions started with a breathalyser test (Lion 500 portable Alcometer; Lion Instruments, UK) upon arrival to ensure a reading of 0.00 prior to testing. All participants produced this reading.

On day 1, after completing the baseline questionnaires (BDI, BIS/BAS and the PM Scale), participants underwent the relevant retrieval procedure according to random allocation (see Fig. 1). They then completed the distractor tasks in a fixed order, followed immediately by the reappraisal procedure, rating of the procedure and immediate recall of reappraisals. The ACQ-SF was then completed followed by the alcohol fluency task.

On day 8, participants were re-tested on memory for the reappraisals generated on day 1, again using their original maladaptive appraisals as cues. They then completed the alcohol attentional bias task followed by the TLFB, ACQ-SF and alcohol fluency.

### Statistical analysis

Data was normally distributed and inspection of outliers from boxplots revealed that one participant’s attentional bias data included extreme values (action-outcome group), which were replaced with the next highest/lowest (non-outlier) values. Repeating the analysis with these values removed did not appreciably affect the statistical outcomes for the attentional bias measures.

One-way ANOVA was used to determine the degree to which groups were matched on baseline demographic, alcohol-related and subjective variables assessed on day 1. Repeated, mixed ANOVA, with group as the between- and day as the within-subjects factor, was used to analyse reappraisal recall, alcohol fluency, craving and alcohol consumption. Since the number of neutral words generated in the verbal fluency task was considerably higher than valenced words, fluency data was log transformed (log_10_ (*x*_i_ + 1)) to equalize variance across valences and allow all three levels of the fluency factor to be included in the same parametric model. When sphericity could not be assumed, Greenhouse-Geisser correction was applied, as indicated in adjustments to degrees of freedom. Post hoc pairwise Bonferroni-corrected *t* tests were used in follow-up analyses. The significance level was set at *р* < 0.05, and all reported statistical values are based on two-tailed tests. Data analyses were performed using IBM SPSS 21.

## Results

### Baseline characteristics

There were no group differences in demographics (age, gender, ethnicity and highest education) or baseline drinking-related or trait measures (AUDIT, BDI, BIS/BAS and psychological mindedness; Table 1).

**Table 1:** Baseline demographic and psychometric characteristics of participants (mean ± SD unless otherwise indicated)

	Total (*n* = 47)	Control (*n* = 16)	Omission-PE (*n* = 16)	Value-PE (*n* = 15)	*χ* ^2^/*F* value	*p*
Age	27.0 (9.57)	25.3 (8.5)	27.1 (9.92)	28.7 (10.50)	0.484	.620
Gender, *N* (%)						
Male	29 (61.7)	9 (56.3)	12 (75.0)	8 (53.3)	1.843	.398
Ethnicity, *N* (%)						
White	28 (59.6)	10 (62.5)	7 (43.8)	11 (73.3)	2.900	.235
Highest education, *N* (%)						
GCSEs	2 (4.3)	1 (6.3)	1 (6.3)	0 (0)		
‘A’ levels	19 (40.4)	8 (50.0)	6 (37.5)	5 (33.3)		
Undergraduate	17 (36.2)	4 (25.0)	7 (43.8)	6 (40.0)		
Postgraduate	9 (19.1)	3 (18.8)	2 (12.5)	4 (26.7)		
AUDIT	16.96 (5.37)	17.25 (5.42)	16.93 (4.00)	16.67 (6.78)	0.044	.957
BDI	7.55 (6.74)	6.75 (3.96)	5.63 (3.59)	10.47 (10.23)	2.289	.113
BAS drive	11.83 (2.30)	12.19 (2.71)	12.00 (2.42)	11.27 (1.62)	0.679	.512
BAS fun seeking	1.191 (2.01)	13.13 (2.47)	13.63 (1.70)	12.80 (1.78)	0.658	.523
BAS reward responsiveness	17.72 (1.63)	17.88 (1.41)	18.06 (1.88)	17.20 (1.57)	1.187	.315
BIS	21.17 (3.25)	21.56 (3.92)	20.31 (1.85)	21.67 (3.64)	0.845	.436
Psychological mindedness	130.06 (25.31)	133.50(24.94)	132.13(22.01)	124.20(29.40)	0.592	.557

### Learning and recall of reappraisals

All three groups showed near perfect recall of reappraisals on day 1 (Table 2). A 3 (group) × 2 (day 1, day 8) ANOVA did not indicate a two-way interaction between time and group (*F*(2,44) = 1.479, *p* > 0.2). A significant effect of day (*F*(1, 44) = 26.58, *p* < 0.001, *η*_*p*_^2^ = .377) indicated better (immediate) recall on day 1 than (delayed) recall on day 8, as expected. A trend-level effect of group was also seen (*F*(2, 44) = 2.626, *p* = 0.084, *η*_*p*_^2^ = .107), indicating somewhat higher overall recall in the value-PE than the control group.

**Table 2:** Memory performance (recall of reappraisals and verbal fluency) by group (mean ± SD)

	Control		Omission-PE		Value-PE	
	Day 1	Day 8	Day 1	Day 8	Day 1	Day 8
Reappraisal recall	96.88 (6.72)	78.13 (19.92)	97.92 (5.69)	84.38 (17.71)	98.89 (4.30)	91.11 (12.39)
Alcohol fluency^a^						
Neutral words	17.88 (7.66)	19.41 (7.65)	15.34 (6.17)	17.25 (6.21)	13.63 (7.23)	14.50 (6.50)
Positive words	1.06 (1.30)	1.47 (2.01)	1.50 (2.03)	0.47 (0.81)	2.43 (2.93)	2.27 (3.33)
Negative words	1.31 (1.82)	0.75 (1.18)	1.41 (1.73)	0.91 (1.68)	1.33 (1.89)	2.23 (2.40)

^a^Untransformed values

### Alcohol fluency

There was a main effect of valence on the verbal fluency task (*F*(1.58,34.20) = 201.72, *p* < 0.0001, *η*_*p*_^2^ = 0.821), with a larger number of neutral words produced across groups on both days compared to valenced words (see Table 2; untransformed values). Trend-level interactions were found between day and group (*F*(2,44) = 3.00, *p* = 0.060, *η*_*p*_^2^ = 0.120) and day and valence (*F*(2, 88) = 2.90, *p* = 0.061, *η*_*p*_^2^ = 0.062). However, there was also a significant three-way interaction between day, group and valence (*F*(2, 88) = 2.56, *p* = 0.044).

**Fig. 2:**
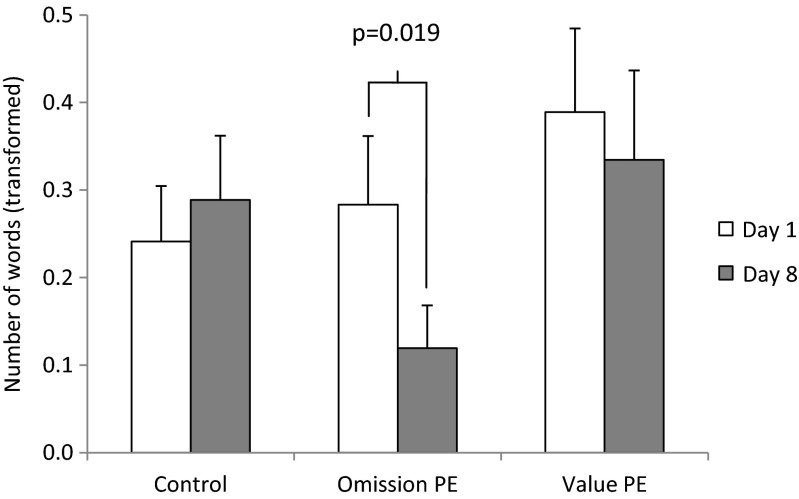
Alcohol fluency. Verbal fluency for positive alcohol-related words across sessions (days 1 and 8) by group. *Bars* are mean values ± SEM of log-transformed values

Between-group pairwise Bonferroni-corrected comparisons conducted at each level of valence and day yielded no significant effects (all *p* values >0.1). In contrast, comparisons between day 1 and day 8 at each level of valence (neutral, positive and negative) and group revealed a significant decrease in the number of positive words in the omission-PE group (*p* = 0.019) (Fig. 2). There were also trends towards an increase in negative words in the value-PE group (*p* = 0.071) and a reduction in negative words at day 8 in the omission-PE group (*p* = 0.076).

### Craving

A three-way (group × day × ACQ-SF subscale) ANOVA showed only a main effect of scale (*F*(3,132) = 35.00, *p* < 0.0001), with higher levels of ‘purposefulness’ craving across groups on both days. Given our previously observed effects on craving following counter-conditioning (Das et al. 2015a) and relatively low power to detect a three-way interaction, exploratory analysis was conducted using two-way (group × day) ANOVAs for each ACQ-SF subscale. This showed an interaction between day and group on the purposefulness subscale of the ACQ-SF (*F*(2, 44) = 3.272, *p* = .047, *η*_*p*_^2^ = .129) but not the other craving subscales (*F* values <1; *p* values >0.1, *η*^2^ values ≤0.033). Pairwise, Bonferroni-corrected post hoc tests examining effects of day showed a significant reduction in craving between day 1 and day 8 in the omission-PE group on the purposefulness subscale (from 3.85 ± 1.36 to 3.06 ± 1.01; *p* = 0.020) but not the control group (3.69 ± 1.09 to 4.04 ± 1.27) or the value-PE group (3.51 ± 1.36 to 3.56 ± 1.36). There was also a trend towards a group effect, such that the omission-PE group had lower overall purposefulness-craving levels than the control group (*p* = 0.058).

### Alcohol consumption

A main effect of day reflected a reduction in alcohol consumption from day 1 (45.47 drinks) to day 8 (37.32 drinks) (*F*(1, 44) = 21.442, *p* = 0.001, *η*_*p*_^2^ = .328). However, there was no interaction between day and group (*F*(2, 44) = 0.756, *p* = 0.476).

### Attentional bias

A 3 (group) × 4(picture type) mixed ANOVA indicated a main effect of picture type (*F*(3, 129) = 3.004, *p* = 0.033, *η*_*p*_^2^ = .065), reflecting higher attentional bias (dwell time) to each of the three alcohol pictures types (*t* (45) = 2.468, *p* = .017; across the three groups and three alcohol picture types compared to neutral). However, there was no interaction between picture type and group (*F*(6,129) = 0.330, *p* = 0.92).

### Readiness-to-change drinking

A 3 (group) × 2 (day) × 3 (subscale) mixed ANOVA on SOCRATES scores showed main effects of day (*F*(1, 44) = 5.466, *p* = 0.024, *η*_*p*_^2^ = .11) and subscale (*F*(2, 88) = 212.747, *p* < 0.001, *η*_*p*_^2^ = .829), subsumed under a day × subscale interaction (*F*(2, 88) = 32.489, *p* < 0.001, *η*_*p*_^2^ = .425). The simple effect of day was significant only for the recognition (*F*(1, 46) = 48.816, *p* < 0.001, *η*_*p*_^2^ = .515) and ambivalence (*F*(1, 46) = 20.65, *p* < 0.001, *η*_*p*_^2^ = .31) subscales indicating increased recognition of drinking-related problems as well as reduced ambivalence about drinking regardless of group.

### Predictors of response

Overall, participants found the reappraisal procedure moderately and equivalently helpful across groups (mean helpfulness rating = 6.94 ± 1.65 across groups (*F*(2,46) = 1.138, *p* = 0.330)). Similarly, there was no difference between groups on how ‘easy’ they found the procedure (mean rating across groups = 6.55 ± 2.60; (*F*(2, 46) = 0.945, *p* = 0.40)). Exploratory correlations found that participants’ self-rating of how helpful they found the reappraisal predicted how many alcohol-related words they recalled on day 8, with those finding the intervention more helpful recalling fewer alcohol-related words (*r*(47) = −.518, *p* = 0.003). Conversely, those who reported finding the reappraisal procedure easier recalled more alcohol-related words on day 8 (*r*(47) = 0.519, *p* = 0.003). This latter association may indicate lower engagement with the procedure and therefore more superficial and ‘easy’ responses given.

### Expected and actual pleasantness of drinks

As participants in the omission error group did not consume any drink during retrieval, it was not possible to assess expected and *actual* pleasantness of the drink in this group. Therefore, a two-way group (control/value-PE group) × time (pre-drink/post-drink) ANOVA was conducted, which indicated a significant interaction on (expected versus experienced) pleasantness ratings of drink (*F*(1,28) = 15.069, *p* = 0.001), consistent with a larger difference in pre-post pleasantness ratings in the control group (mean pre-post difference 5.44 ± 2.29) than the value-PE group (mean difference = 2.00 ± 2.42).

## Discussion

In this study, we examined the effects of a prototypical cognitive behavioural emotion regulation procedure—reappraisal (of maladaptive drinking-related appraisals)—on alcohol-related memory parameters, as well as non-memory outcomes (e.g. craving, attentional bias, drinking behaviour). Critically, we examined these effects within the context of two different procedures designed to destabilize alcohol memory. We found partial support for memory updating following reappraisal in the context of memory labilization on one of the two primary memory-related measures (alcohol fluency). Specifically, performance on verbal fluency for positively valenced alcohol-related words was impaired in the omission-PE group. We interpret this effect to reflect changes in the semantic structures that support rules and schemas related to alcohol use, particularly positive alcohol appraisals. No effects were found on memory for reappraisals themselves. However, it is worth noting that the combination of excellent inter-rater agreement on scoring of accuracy for memory reappraisals, high levels of immediate recall and moderate levels of perceived helpfulness of the reappraisal procedure suggest that this is a robust and reliable method that may translate well to other studies examining memory for psychotherapeutic ‘components’ (Harvey et al. 2014).

Effects on the purposefulness subscale of the ACQ-SF in the omission-PE group must be considered with caution given the lack of an a priori hypothesis specifically related to this subscale and the lack of a three-way interaction involving the ACQ-SF subscale factor. In addition, there were no group differences on attentional bias to alcohol (cf. Das et al. 2015a) suggesting that such effects depend on the nature of the manipulation carried out within the reconsolidation window. In our previous study on counter-conditioning (Das et al. 2015a), the aim had been to change valuation of alcohol through relatively basic associative learning processes, and the outcomes were chosen in line with that aim. Similar associative learning-related outcomes (e.g. conditioned fear responses or ‘cue reactivity’) have been used in studies of retrieval-extinction, another intervention which involves simple associative learning (e.g. Schiller et al. 2009; Xue et al. 2012). Here, our primary outcomes were also selected to match our aims, which focused on introducing alternative episodic and semantic rather than associative information. The lack of effect on secondary outcomes suggests that reappraisal carried out within the reconsolidation window does not result in a comprehensive and generalized restructuring of alcohol memory networks.

Our specific test of the effects of reappraisal in the context of memory destabilization/reconsolidation was motivated by the goal of developing procedures for improving the efficacy of psychological treatments of addictive disorders. As noted by Harvey and colleagues, the efficacy of psychosocial treatments could potentially be enhanced if patients were better able to recall the contents of their treatment sessions (Harvey et al. 2014). Given its emphasis on the development of new (or modifying old) rules and schemas, the effectiveness of CBT in particular is likely to be improved by better memory for instances of emotion regulation, such as specific reappraisals of maladaptive rules generated within a treatment session. We believe that the current study is the first to test declarative memory for therapeutically meaningful, self-generated emotional regulation material in the context of memory destabilization. Although we do not find statistical support for improved memory in the PE groups, it is worth noting that both PE groups showed higher reappraisal memory on day 8 compared to the control group. For example, while the value-PE group showed only ∼8 % reduction in recall of reappraisals by day 8, there was a ∼19 % reduction in the control group. As such, we believe that this remains an important area for future investigation.

In a related vein, the current study raises a number of considerations for testing different PE-generating/memory-destabilizing procedures in humans (Das et al. 2015a). Accepting the necessity of PE in memory destabilization (Lee 2009; Pedreira et al. 2004; Sevenster et al. 2013b), it seems likely that the application of reconsolidation-modulation to modify or weaken maladaptive memory traces in clinical disorders will rely on identifying a *variety* of PE-memory destabilization procedures, since any given PE-generating procedure would likely become ineffective (no longer surprising) after a single use.

While we assessed pleasantness of drinks, we had no independent and specific measure of PE (e.g. subjective ratings of surprise after omission or devaluation of beer). As such, we cannot make strong claims that the value-PE condition (or indeed the omission-PE condition) actually produced a PE. Moreover, we do not know whether the value- and omission-PE conditions were equivalent to each other in terms of degree of mismatch between expectation and outcome. For example, it is possible that the level of surprise in the value-PE condition was insufficient to cause memory destabilization. Indeed, prior to the experiment, participants would have been aware that they may be required to drink bitter-tasting liquids (this was disclosed in the study information-sheet), whereas they would not have known that alcohol might be withheld at the last moment. It is possible therefore that there was a reduced discrepancy between expectation and outcome in the value-PE condition which rendered it less effective. Future studies should gauge expectancy violations through subjective ratings of surprise following PE-generating procedures.

Another potential explanation for an absence of statistically significant effects of value-PE is that our participants had strong pre-existing S-R associations supporting habitual responding, which is relatively impervious to outcome value degradation (Ostlund and Balleine 2009). However, while our participants were selected to show problematic drinking patterns, dependent participants—those most likely to show habitual responding and less sensitivity to changes in value of the outcome—were specifically excluded from our study. Nonetheless, the interaction between expectancy violation (PE), putative memory destabilization and the tendency to respond habitually to alcohol cues is an important one to consider in future tests of value-PE in the context of memory destabilization.

This study was intended as a proof-of-principle experiment, and some limitations should be acknowledged. Firstly, the sample was relatively small, which may have resulted in small effects being obscured. A better-powered study would help us clarify the importance of some currently suggestive (though statistically non-significant) findings, like those outlined above.

Secondly, while we tested two PE procedures, we only had one control group, which was more closely matched to the value- than the omission-PE group. While it seems highly unlikely that simple retrieval of non-alcohol memories and non-alcohol-related reward omission (i.e. an orange juice omission group) would have been sufficient to cause effects on alcohol memory, this possibility could be excluded through the use of appropriate additional control groups in future studies. However, it should be noted that as reconsolidation-updating effects are deduced from patterns of differences between groups, this poses a problem in general for reconsolidation interference research. In order to conclusively infer that a result was due to a reconsolidation manipulation and not various non-specific factors, effects must be shown to be retrieval- *and* intervention-dependent. Factorially, this requires four groups in basic studies of reconsolidation. Attempts to control for non-specific effects such as increased arousal following a reminder procedure require additional controls. Such experiments quickly become unwieldy, requiring very large sample sizes even for modest Ns per group. An ability to independently and reliably measure memory destabilization when it occurs would overcome this and many related epistemological problems in the area and should therefore be considered an area of research priority.

Third, by way of proviso, it should be noted that this study was mechanistic and was not intended to model the full richness and complexity of the psychotherapeutic encounter. The latter relies on the activation of emotion (emotional processing) and the therapeutic relationship, as well as the application of specific techniques, such as reappraisal. In their account of the role of memory reconsolidation in psychotherapy, Lane and colleagues (Lane et al. 2014) contend that these other ingredients are essential to the transformative effects of psychotherapy. It is therefore of interest to determine the added value of PE-retrieval/reconsolidation in the context of a formal psychological treatment.

Despite these methodological limitations, our findings are in line with the hypothesis that PE-generated memory instability can allow alcohol memories to be modified. We add to the existing literature on retrieval-extinction (Xue et al. 2012) and post-retrieval counter-conditioning (Das et al. 2015a) by showing that reappraisal has promise as a post-retrieval strategy for reducing the influence of maladaptive memories in substance use disorders. It remains of interest to determine which other behavioural (and pharmacological) procedures can be added to this list. It may be that the development of reconsolidation-modulation-based therapies will rely on a suite of such techniques or the consolidated application of one or a few of those shown to be most effective. In addition, the choice of procedure may depend on individual participant (patient) characteristics. For example, individuals who are highly cue reactive might benefit most from a retrieval-extinction intervention, whereas those who have excessively high evaluative representations of alcohol may benefit more from retrieval counter-conditioning or the retrieval-reappraisal reported here. Ongoing research in this area will help to clarify the conditions under which reconsolidation manipulations might enhance efficacy of treatments for alcohol and substance use problems.
